# Supplementary material for: Original Leaf Colonisers Shape Fungal Decomposer Communities of Phragmites australis in Intermittent Habitats
Source: J Fungi (Basel). 2022 Mar 10;8(3):284. doi: 10.3390/jof8030284 (PMC8951327; doi:10.3390/jof8030284)
Supplement: Supplementary file 1 [file jof-08-00284-s001.zip › jof-1620659-supplementary.pdf]

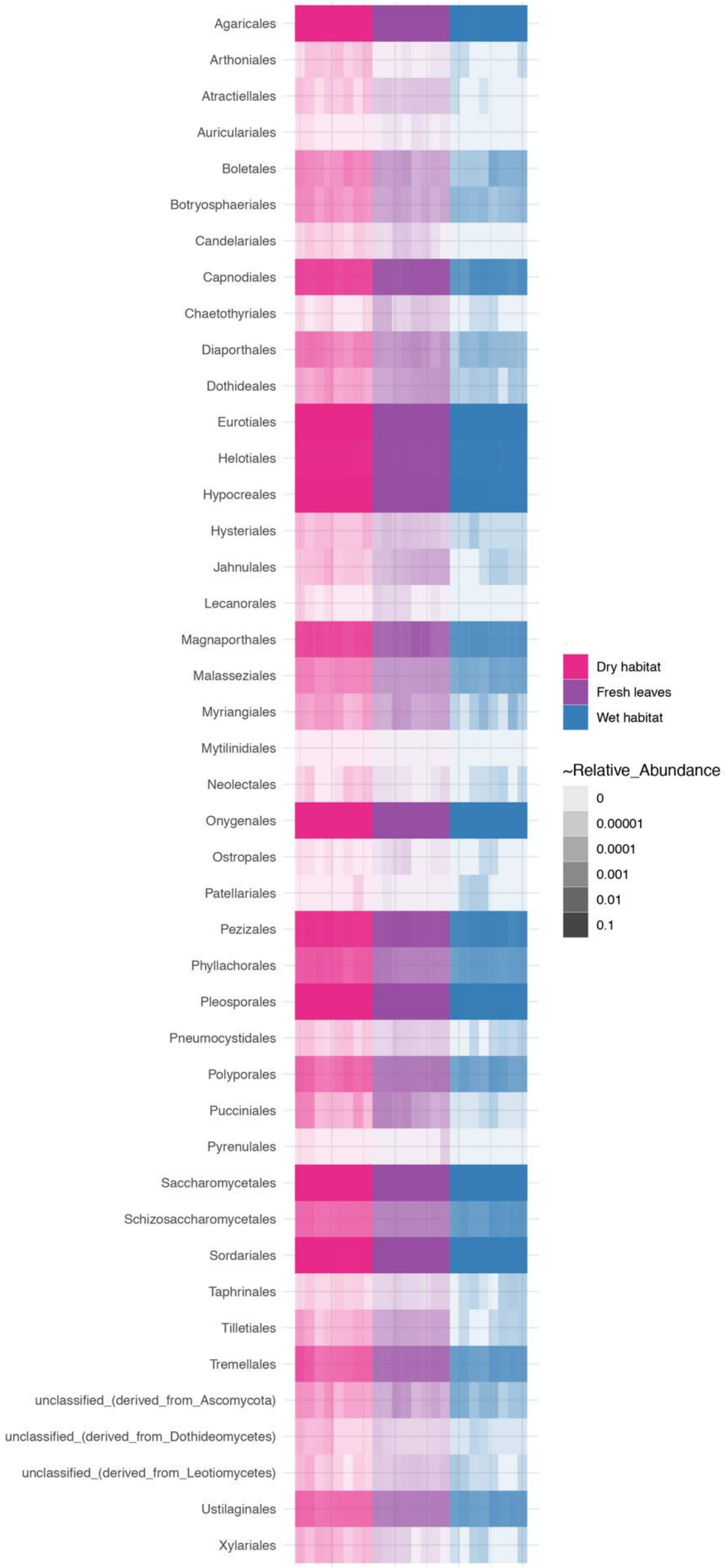

Supplementary Figure S1. Scaled relative abundance of all OTUs from the fresh leaves and leaves decomposing in either wet or dry habitat. Individual tiles represent the individual samples (total N = 24).

Supplementary Table S1. Node degrees for keystone OTUs for individual location's meta-networks, defined as nodes within the top 5% of node degree values of each network.

	**Fresh leaves**	**Dry habitat**	**Wet habitat**
Ajellomyces			18
Arthroderma	20		
Aspergillus			18
Botryotinia	20		
Chaetomium			18
Coccidioides			18
Gibberella	20		
Magnaporthe			18
Neurospora			18
Podospora			18
Talaromyces	20	21	18
Uncinocarpus	20		18
Verticillium			18
